# Mechanical Properties of Composite Hydrogels of Alginate and Cellulose Nanofibrils

**DOI:** 10.3390/polym9080378

**Published:** 2017-08-19

**Authors:** Olav Aarstad, Ellinor Bævre Heggset, Ina Sander Pedersen, Sindre Hove Bjørnøy, Kristin Syverud, Berit Løkensgard Strand

**Affiliations:** 1 NOBIPOL, Department of Biotechnology and Food Sciences, NTNU Norwegian University of Science and Technology, NO-7491 Trondheim, Norway; olav.a.aarstad@ntnu.no (O.A.); inasande@stud.ntnu.no (I.S.P.); 2 RISE PFI, Nanocellulose and carbohydrate polymers, Høgskoleringen 6b, 7491 Trondheim, Norway; ellinor.heggset@rise-pfi.no (E.B.H.); kristin.syverud@rise-pfi.no (K.S.); 3 Department of Physics, NTNU Norwegian University of Science and Technology, NO-7491 Trondheim, Norway; sindre.bjornoy@ntnu.no; 4 Department of Chemical Engineering, NTNU Norwegian University of Science and Technology, NO-7491 Trondheim, Norway

**Keywords:** alginate, TEMPO, cellulose nanofibrils, nanocellulose, composite, hydrogels, mechanical properties

## Abstract

Alginate and cellulose nanofibrils (CNF) are attractive materials for tissue engineering and regenerative medicine. CNF gels are generally weaker and more brittle than alginate gels, while alginate gels are elastic and have high rupture strength. Alginate properties depend on their guluronan and mannuronan content and their sequence pattern and molecular weight. Likewise, CNF exists in various qualities with properties depending on, e.g., morphology and charge density. In this study combinations of three types of alginate with different composition and two types of CNF with different charge and degree of fibrillation have been studied. Assessments of the composite gels revealed that attractive properties like high rupture strength, high compressibility, high gel rigidity at small deformations (Young’s modulus), and low syneresis was obtained compared to the pure gels. The effects varied with relative amounts of CNF and alginate, alginate type, and CNF quality. The largest effects were obtained by combining oxidized CNF with the alginates. Hence, by combining the two biopolymers in composite gels, it is possible to tune the rupture strength, Young’s modulus, syneresis, as well as stability in physiological saline solution, which are all important properties for the use as scaffolds in tissue engineering.

## 1. Introduction

Recently, composite materials of alginate and cellulose nanofibrils (CNF) have shown promising results for bioprinting and tissue engineering applications [1,2,3]. In particular, the shear thinning properties of CNF combined with the viscous alginate that form hydrogels with divalent cations at physiological conditions, are attractive for bioprinting [1].

Alginates are linear copolymers of 1 → 4 linked β-d-mannuronic acid (M) and α-l-guluronic acid (G). The monomers are arranged in a block-wise pattern along the chain with homopolymeric regions of M and G termed M- and G-blocks, respectively, interspaced with regions of alternating structure (MG-blocks) [4]. Alginate forms hydrogels by crosslinking with divalent cations where, particularly, the G-blocks, but also the MG-blocks, are important for the mechanical properties of the resulting gel [5,6]. Alginate hydrogels can be produced under physiological conditions [7,8]. This, together with a low immunogenic profile [9], makes them popular materials for biomedical applications and, in particular, in tissue engineering [10]. Cellulose provides structural support in plant cell walls and can be processed as fibres or nanoscaled fibrils of cellulose, known as cellulose nanofibrils (CNF). Several procedures for preparation of CNF from cellulose pulp exist, among them 2,2,6,6-tetramethylpiperidine-1-oxyl radical (TEMPO)-mediated oxidation using sodium hypochlorite as oxidant [11]. By this, aldehyde and carboxyl groups are introduced on the surfaces of the cellulose fibrils and increase the negative charge of the fibrils. Aqueous dispersions of CNF have gel like character at low concentrations (approx. 0.5%) held together by fibril entanglement and hydrogen bonds. CNF hydrogels can be produced with cations, both monovalent and with higher valency [12], where gels with higher valency ions form stronger gels [12,13].

Due to their availability, renewability, biocompatibility, and low toxicity, both alginate and cellulose are attractive materials for a range of applications such as films, gels, and as viscosifiers. Although being suggested for tissue engineering applications, not much is known about the mechanical properties of composite gels of alginate and CNF. Composite gels of alginate and either oxidized CNC (cellulose nanocrystals) or CNF have shown increased compression strength, suggested to be the result of Ca^2+^ mediated crosslinking of the two components [14]. TEMPO-oxidized BNC (bacterial nanocellulose) have been used to improve the mechanical and chemical stability of an alginate hydrogel, and the composite gel was used for encapsulation of fibroblasts. The nanofibrous structure of the BNC was suggested to mimic the fibre structures of collagen and fibronectin found in the extracellular matrix [15,16,17], and fibroblast viability and proliferation was found to be higher in comparison to pure alginate gels [18]. For chondrocytes, the combination of sulfated alginate with nanocellulose were promising regarding cell viability and phenotype [2]. It has previously been shown that Young’s modulus of the matrix is important for the development of stem cells into differentiated cell types/tissues [19]. Hence, the structure, chemistry, as well as the mechanical properties are of importance in tissue engineering applications. Although composite gels of CNF and alginate have been demonstrated in the literature, a systematic study of mechanical properties on combinations of different types of alginate and CNF has not yet been reported.

We hypothesize that by combining alginate and CNF, the advantageous properties of the individual constituents, i.e., the stiffness of CNF and the compressibility of alginate, could be preserved in the composite gel making it possible to tailor the mechanical properties.

## 2. Materials and Methods

### 2.1. Materials

**Alginates**: Alginates extracted from *Durvillea potatorum* and *Laminaria hyperborea* stipe were obtained from FMC Health and Nutrition (Sandvika, Norway), and *Macrocystis pyrifera* alginate were purchased from Sigma-Aldrich (St. Louis, MO, USA). Molecular weight and NMR parameters are given Table 1. For a detailed study on alginate fine structure see [20].

**Nanocellulose**: Never-dried bleached kraft softwood pulp fibres were used as the source material for production of two types of nanocellulose. The first type was produced using a mechanical pretreatment (beating in a Claflin mill; 1000 kWh/ton for 1 h) followed by homogenization (denoted as mechanically-fibrillated CNF). Production of the second type (referred to as oxidized CNF) was performed using TEMPO-mediated oxidation as pretreatment, as described by Saito and colleagues [11]. NaClO (2.3 mmol per gram of cellulose) was used in the oxidation. The fibrillation was done by using a Rannie 15 type 12.56x homogenizer (APV, SPX Flow Technology, Silkeborg, Denmark) with a pressure drop of 1000 bar in each pass. Mechanically-fibrillated CNF was homogenized using five passes, the oxidized CNF using 1 pass. The concentration of the pretreated samples was 1% (*w*/*v*) before fibrillation.

The composition of carbohydrates was determined according to the standard method NREL/TP-510-42618, using sulphuric acid hydrolysis. The composition of carbohydrate monomers produced during the hydrolysis was analysed using high-performance anion-exchange chromatography with pulsed amperometric detection (HPAEC-PAD; Dionex ICS-5000, (Thermo Fisher Scientific, Waltham, MA, USA) with a CarboPac PA1 column (Thermo Fisher Scientific, Waltham, MA, USA) and gradient elution using sodium hydroxide for elution. The carbohydrate composition of the two CNF samples is shown in Appendix A.

The intrinsic viscosity was determined as described in ISO-standard 5351:2010, using cupriethylenediamine (CED) as a solvent. The oxidized CNF was subjected to a second oxidation before the analysis. This is due to a significant amount of aldehyde groups in the oxidized samples which induce depolymerization through a β-elimination reaction [21]. In order to avoid this, a selective oxidation of the aldehyde groups was performed using sodium chlorite under acidic conditions.

The carboxylate content was determined using conductometric titration as previously described [22,23]. The equipment used was a 902 Titrando, an 856 conductivity module and Tiamo software (Metrohm). Residual fibres were quantified with a FibreTester device, as previously described by Chinga-Carrasco and colleagues [24]. The fibrillated structure and analyses of the fibre structure from atomic force microscopy (AFM) imaging with resulting characteristics of the cellulose nanofibrils have previously been described [25]. A summary of nanocellulose characteristics is shown in Appendix A.

**Control polymers**: Hyaluronic acid (*M*_w_ = 8.7 × 10^5^ Da) was a gift from Novamatrix (Norway)*,* xanthan (*M*_w_ = 9.6 × 10^5^ Da) was a gift from CP Kelco (USA), and dextran (*M*_w_ = 2.0 × 10^6^ Da) was purchased from Pharmacia AB (Stockholm, Sweden). The molecular weight of the samples were determined using a HPLC system consisting of serially connected TSK G6000 PWxl and G5000PWxl columns (Tosoh Bioscience LLC, Tokyo, Japan) followed by a Dawn Helios II MALLS photometer and an Optilab T-rEX differential refractometer (Wyatt Technology, Santa Barbara, CA, USA). The elution buffer (0.15 M NaNO_3_ with 1 mM EDTA, pH 6.0) was applied at a flow rate of 0.5 mL/min. Data were collected and processed using ASTRA 6.1 software. Refractive index increment values (dn/dc) used in the calculations were set to 0.148 for dextran and 0.150 for alginate, hyaluronic acid and xanthan.

### 2.2. Preparation of Gel Cylinders

**Alginate gels** were prepared by internal gelling with CaCO_3_ and d-glucono δ-lactone (GDL) as previously described [26]. Briefly, CaCO_3_ (56.25 mg, particle size 4 µm) suspended in 5 mL MQ water was added to 25 mL, 1.5% (*w*/*v*) of alginate solution. The mixture was degassed under vacuum suction before addition of 7.5 mL of a freshly made solution of GDL (200.36 mg). The mixture was immediately poured into 24-well tissue culture plates (16/18 mm, Costar, Cambridge, MA, USA) and left at room temperature for minimum 20 h. The gel cylinders were removed from the tissue culture plates and weight was measured on the calcium unsaturated gels before being immersed in a solution of 50 mM CaCl_2_ and 200 mM NaCl (100 mL per gel) and left for 24 h at 4 °C for calcium saturation before syneresis and compression measurement.

**Two-component gels** of alginate and either xanthan, dextran or hyaluronic acid were made by a simillar procedure as for pure alginate gels. The two components were dissolved together in 25 mL MQ water, yielding a final concentration in the gelling solution of 1% (*w*/*v*) *M. pyrifera* alginate and 0.30% or 0.75% (*w*/*v*) of the other component.

**Alginate—CNF composite gels** were made by mixing 450 mg alginate powder with MQ water and CNF dispersion corresponding to final concentrations of 1% (*w*/*v*) alginate and 0.15–0.75% (*w*/*v*) CNF and a total volume of 30 mL. The blends were dissolved for at least 18 h under constant stirring and homogenized for 5 min with a Vdi 12 Ultra Turrax at 11,500 rpm. Twenty-five grams of the blend was weighed out and added CaCO_3_ and GDL as described above.

### 2.3. Syneresis and Gel Strength Measurements

Syneresis was determined after calcium saturation as (W_0_ − W)/W_0_) × 100, where W_0_ and W are the initial weights of the samples before calcium saturation and final weights of the calcium saturated gels, respectively. Force/deformation curves was recorded at 22 °C on a Stable Micro Systems TA-XT2 texture analyser equipped with a P/35 probe and at a compression rate of 0.1 mm/s [26]. The gels were subjected to uniaxial compression surpassing the point of rupture, where force and deformation was recorded. Young’s modulus was calculated as G·(h/A) where G is the initial slope (N/m) of the stress deformation curve and h and A is the height and area of the gel cylinder, respectively. In order to compare Young’s modulus of gels with different degree of syneresis, the relationship E = k·c^2^ was applied [27] where k is characteristic for each type of alginate, and c is the weight concentration.

### 2.4. Volume Stability and Calcium Binding

The weight and calcium content of pure and composite gels of *M. pyr.* alginate and oxidized CNF were measured before and after saline treatments. Gel cylinders immersed in a solution of 50 mM CaCl_2_ and 200 mM NaCl for 24 h were cut in three approximately equal pieces, weighed and immersed separately in 40 mL of 150 mM NaCl solution at room temperature for 24 h. The gel pieces were gently removed from the solution, weighed and subjected to a second saline treatment. After weighing, the gels were added 0.5 mL of 100 mM Na-EDTA, pH = 7.0. All samples were vortexed and centrifuged (2400 rpm, 5 min) before they were diluted 100 × in 5% HNO_3_ and calcium content analysed with ICP-MS (type 8800 Triple Quadrupole ICP-MS with ASX-520 Autosampler from Agilent Technologies, Santa Clara, CA, USA). As internal standards ^115^In and ^89^Y were used.

### 2.5. Light Microscopy

Homogeneity of the gels were assessed on a Nikon Eclipse TS100 microscope (Nikon Instruments, Tokyo, Japan) by placing a drop of gelling solution between two microscopy slides and observing the gellation in the microscope using 40 × magnification.

### 2.6. Preparation of Aerogels for Scanning Electron Microscopy (SEM)

After the gels had been cast, the cylinders where cut, in the hydrated state, into 200 µm thick sections using a vibrating blade microtome (VT1000S, Leica Biosystems, Nussloch GmBH, Wetzlar, Germany). The sections where dehydrated in increasing concentrations of ethanol, before substitution with acetone and finally critical-point dried using liquid CO_2_ (Emitech K850 critical point dryer, Quorum Technologies, Lewes, UK). The sections were mounted on aluminum stubs using carbon tape and sputter coated (208 HR, Cressington, Watford, UK) with a 5 nm layer of Pt/Pd (80/20). SEM analysis was performed with an acceleration voltage of 5 kV (S-5500 S(T)EM, Hitachi, Tokyo, Japan).

### 2.7. Fourier Transform Infrared Spectroscopy (FTIR) Characterization

The infrared spectra of vacuum dried samples were collected on a Bio-Rad Excalibur series FTS 3000 spectrophotometer (Bio-Rad, Hercules, CA, USA). The spectra were acquired in transmission mode on the films at a spectral range of 4000−500 cm^−1^.

### 2.8. Statistical Analysis

All values are expressed as means ± standard deviations. Comparisons between groups were made using a two-sided Student’s t-test and Microsoft Excel worksheet (2011). Statistical outcomes (*p*-values) are presented in Appendix B.

## 3. Results and Discussion

### 3.1. Composite Hydrogels of Alginate and CNF

**Figure 1:**
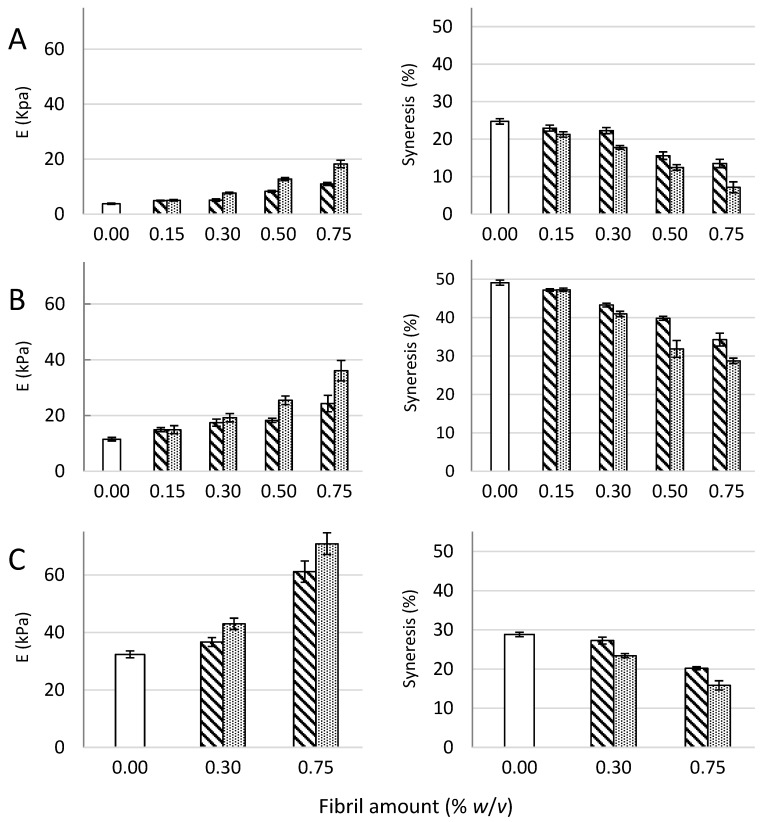
Young’s modulus (E, left panel) and syneresis (right panel) of alginate-CNF composite Ca-gels made from 1% (*w*/*v*) *D. potatorum* (**A**), *M. pyrifera* (**B**), and *L. hyperborea* (**C**) alginates and 0–0.75% (*w*/*v*) mechanically-fibrillated (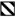) and oxidized CNF (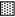). Bars are means ± standard deviations, shown for *n* = 5–8 gels. *P*-values are presented in Table A3 and Table A4 in Appendix B.

**Figure 2:**
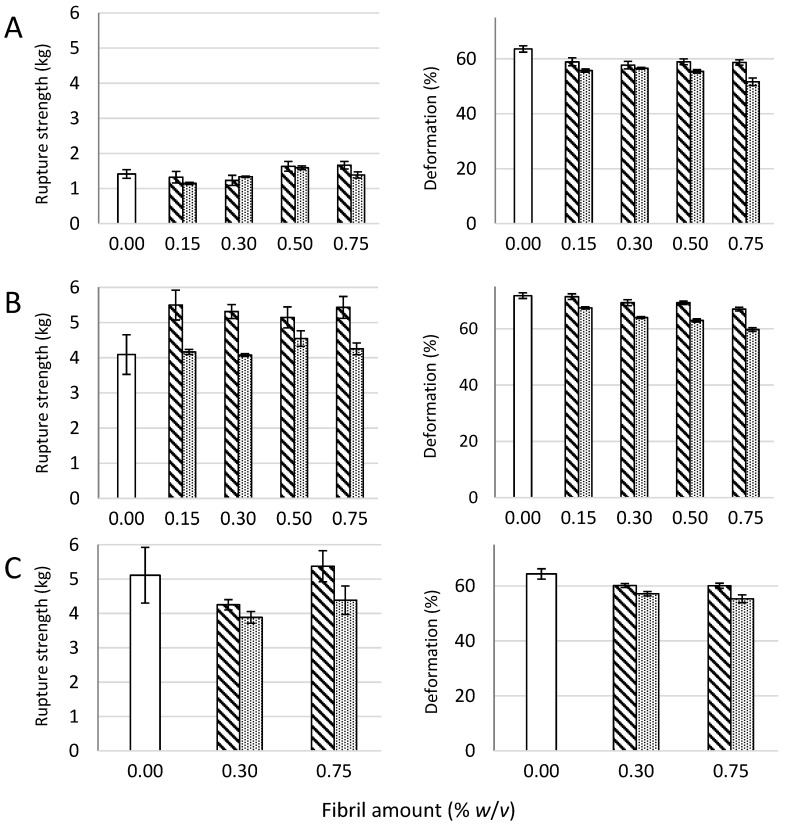
Rupture strength measured as the force at rupture (left panel) and compression at rupture (right panel) for *D. potatorum* (**A**), *M. pyrifera* (**B**), and *L. hyperborea* (**C**) Ca-alginate gels and mechanically-fibrillated (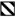) and oxidized (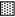) CNF. Bars are means +/− standard deviations, shown for *n* = 5–8 gels. *P*-values are presented in Table A3 and Table A4 in Appendix B.

Composite gels of *D. pot.*, *M. pyr.*, and *L. hyp.* alginates and CNF (mechanically-fibrillated or oxidized) saturated with calcium were measured with respect to gel rigidity (Young’s modulus) and volume reduction upon gel formation (syneresis, Figure 1). Secondly, resistance to breakage and deformation at breakage was determined (Figure 2).

The composite gels of alginate and CNF showed increased Young’s modulus and reduced syneresis compared to pure alginate gels. An increase in Young’s modulus was observed for composite gels of both mechanically-fibrillated CNF and oxidized CNF relative to the alginate gels alone. The largest effect was seen for composite gels of oxidized CNF and alginates with intermediate to low G-content (*M. pyr.* and *D. pot.*) with a 3–5 fold increase in Young’s modulus for the gels containing 0.75% (*w*/*v*) oxidized CNF relative to no addition of CNF. Additionally, for the alginate with a high G-content (*L. hyp.* stipe) that is known to form stiff gels with calcium [27], the effect of addition of CNF on Young’s modulus was profound with about two times increase for the highest concentration. Syneresis was reduced for the composite gels relative to the alginate gels, meaning that the gels shrunk less after saturation with calcium when CNF was added to the hydrogels. As seen from Figure 1, the alginate gels are highly syneretic when saturated with calcium. The syneresis is known to be linked to the content of G-blocks, but indeed also to MG-blocks in the alginate [5,26], hence, the high degree of syneresis for the alginate from *M. pyr.* is as expected. Regardless of the composition of the alginate, CNF reduced the syneresis with increasing effect at increasing concentrations. Although the reduction in syneresis was highest for the oxidized CNF—*M. pyr.* alginate composite gel (49% to 29%), the largest relative decrease was observed for *D. pot.* alginate where the syneresis was reduced from 25% to 7% with the addition of 0.75% (*w*/*v*) oxidized CNF.

No significant effect of the presence of fibrils was seen on the rupture strength of the gels, except for the increase in rupture strength for the gels of alginate from *M. pyr.* upon the addition of mechanically-fibrillated CNF (Figure 2). An overall trend was a slight reduction in the rupture strength when oxidized CNF was added to the alginate relative to the mechanically-fibrillated CNF. However, the alginate concentration is higher in the pure alginate gels than in the composite gels, due to the large differences in syneresis. The rupture strength per concentration unit alginate is, therefore, increased in the composite gels. Calcium alginate gels with a high content of G-blocks, such as the alginate from *L. hyp* stipe, are known to form strong gels due to the high numbers of crosslinks, as also reflected in the Young’s modulus (Figure 1). Alginate from *M. pyr.* has a high content of MG-blocks and is known to withstand large deformation. This has been explained in terms of energy dissipating effects of collapsing MG block junction zones during deformation [28]. Surprisingly, the force at rupture increased by about 30% when mechanically-fibrillated CNF was added to this alginate gel, independently of the concentration of fibrils.

### 3.2. Composite Hydrogels of Oxidized CNF and Alginate

**Figure 3:**
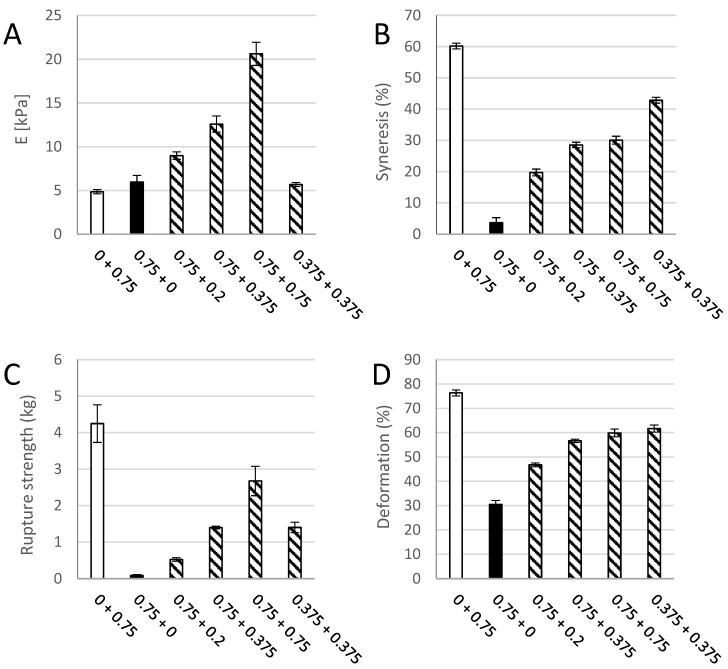
Young’s modulus (E, (**A**)), syneresis (**B**), rupture strength (**C**) and deformation at rupture (**D**) of a pure 0.75% (*w*/*v*) *M. pyrifera* alginate gel (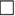), a pure (0.75% *w*/*v*) oxidized CNF gel (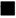), and composite gels of 0.75% (*w*/*v*) of each of the two materials (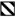). Mean +/− standard deviation shown for *n* = 5–8 gels.

In order to better evaluate separate and combined effects of the two biopolymer components, calcium crosslinked gels with either oxidized CNF or alginate solely (0.75% (*w*/*v*)) were prepared and measured with respect to gel rigidity, syneresis, deformation, and force at rupture (Figure 3). Hydrogels of TEMPO-oxidized CNF have previously been produced with both monovalent and divalent ions [12]. Although the Young’s modulus of alginate and oxidized CNF gels were similar in our study, large differences were seen for the syneresis and the rupture strength underlining the contribution of the two components to the composite system. Oxidized CNF gels displayed almost no syneresis in contrast to the highly syneretic gels from *M. pyr.* alginate. For the rupture strength, the alginate contributed with a high degree of compressibility as shown by the compression to 70% before rupture at about 4 kg force. The oxidized CNF, on the other hand, had a rupture strength of less than 0.1 kg. A dose-dependent increase in syneresis and rupture strength upon the addition of alginate to oxidized CNF was observed. The potential effect on the properties by increasing the dry weight content in the hydrogels were studied by a control containing half concentration of the two components and thus same dry matter content as in the systems of alginate and CNF alone. Additionally, here, a combined effect of the two components could be seen on the syneresis and rupture strength. A combination of 0.75% alginate with 0.75% oxidized CNF resulted in a more than four-fold increase in Young’s modulus, exceeding a solely additive effect of the two separate systems. Representative force-deformation curves of composite gels of alginate and oxidized CNF is shown in Appendix D.

### 3.3. Composite Hydrogels of Alginate and Other Polysaccharides

**Figure 4:**
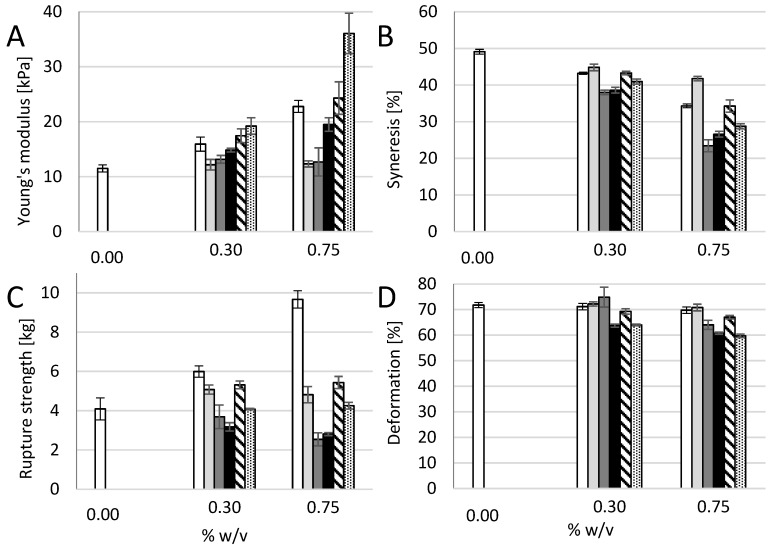
Young’s modulus (E, (**A**)), syneresis (**B**), rupture strength (**C**), and deformation at rupture (**D**) of *M. pyrifera* alginate gels (1% *w*/*v*) with 0.30 and 0.75% (*w*/*v*) addition of *M. pyrifera* alginate (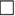), dextran (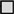), hyaluronic acid (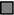), xanthan (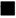), mechanically-fibrillated CNF (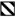) and oxidized CNF (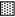). Bars are means +/− standard deviations, shown for *n* = 5–9 gels. *P*-values are presented in Table A5 and Table A6 in Appendix B.

To study more specifically the effect of addition of the long cellulose nanofibrils to alginate, control experiments using other polysaccharides in composite alginate hydrogels were conducted. Polysaccharides with approximately the same molecular weight, but different charge density and flexibility were added to calcium-alginate hydrogels of *M. pyr.* alginate to study the effect on mechanical properties. Dextran, having an α-(1,6)-linked glucose backbone with varying degree of α-(Glc-1,3) branching, is a highly-flexible polysaccharide without charges. Sodium hyaluronate is a stiffer molecule, with alternating 4-linked β-d-glucuronic acid and 3-linked *N*-acetyl-β-d-glucosamine, with negative charges due to the guluronic acid, but with no specific calcium affinity. Xanthan consists of a (1,4)-β-d-Glc main chain where every second unit is (3,1) linked to a d-Man-(1,4)-β-d-GlcA-(1,2)-α-d-Man side chain with varying degree of acetyl and pyruvate groups on the internal and terminal mannose. Xanthan has the ability to form very stiff double-stranded structures. Finally, increasing concentrations of alginate were investigated to compare the effects relative to the effects of addition of CNF on Young’s modulus, syneresis, and resistance to rupture (Figure 4).

Addition of dextran and sodium hyaluronate to the alginate did not affect the Young’s modulus for any concentration showing that neither the increase in dry matter nor net charge by itself result in an increase in gel stiffness. A concentration-dependent increase in stiffness was seen for alginate from *M. pyr*. Although having a relatively high content of MG-blocks, the alginate from *M. pyr.* also contains a fraction of very long G-blocks [20]. Both G-blocks and MG-blocks are known to contribute to the crosslinking with calcium [5], hence, an increase in stiffness upon increase in concentration of alginate for the calcium saturated gels is as expected with increasing numbers of crosslinks per unit volume. For 0.75% (*w*/*v*) added polysaccharides, xanthan, and mechanically-fibrillated CNF gave about a doubling in the Young’s modulus, compared to the pure alginate gel (1% (*w*/*v*)). Xanthan, which can be viewed as rigid rods at the length scale of a G-block junction zone, shows similarities with the addition of long G-blocks to alginate gels that results in stiffer gels with reduced syneresis [29]. The very long G-blocks have been suggested to act as reinforcement bars in the alginate hydrogel network. However, the largest increase was obtained by the addition of oxidized CNF with a 3.5 times increase in Young’s modulus compared to the 1% alginate gel and also a 50% increase in Young’s modulus compared to the 1.75% alginate gel for the composite gel of 1.0% alginate and 0.75% oxidized CNF. This emphasizes the combined effect of both stiffness and length of the fibres combined with the charge being important for the final stiffness of the composite gels.

In general, all the added biopolymers caused a concentration-dependent reduction in syneresis (Figure 4). Least effect was seen for the dextran, which caused slightly more syneresis than increasing the concentration of alginate. The syneresis was markedly reduced by adding xanthan and hyaluronic acid, compared to alginate alone (Figure 4). This suggests that osmotic pressure, rather than interference with G-block junction zones, affects the degree of syneresis for these composite gels. The addition of mechanically fibrillated CNF resulted in same syneresis as for the increased alginate concentrations, however, again the oxidized CNF caused less syneresis.

The rupture strength of the alginate gels was highly influenced by the alginate concentration (Figure 4). By increasing the concentration of alginate from 1.0% to 1.75% (*w*/*v*), the rupture strength increased from 4 kg to 9 kg. None of the other additives were near this increase of rupture strength. Again, there was a slight positive effect on the rupture strength by the addition of mechanically-fibrillated CNF, and no effect was seen on the addition of oxidized CNF although a slight reduction in deformation at rupture could be observed. Hence, ionic interactions between the alginate and cellulose may be weak, although significant, for the initial resistance against compression, however, they are not comparable to the alginate crosslinking when it comes to resisting the force at high deformations. Dextran had a slight positive effect on the rupture strength, however, both hyaluronan and xanthan caused a reduction in the rupture strength.

### 3.4. Composite Gel Morphology and Homogeneity

**Figure 5:**
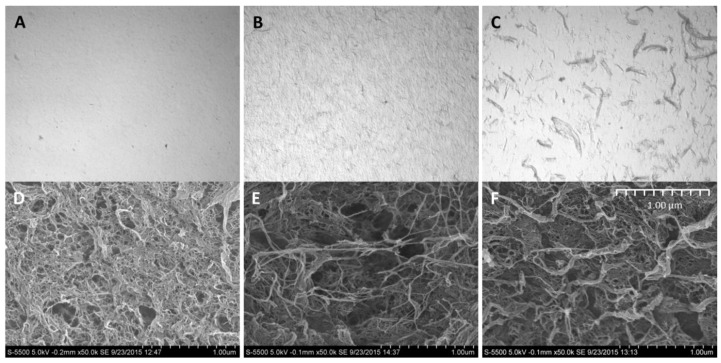
Microscopy of hydrogels of *M. pyrifera* alginate (1% (*w*/*v*)) (**A**), and composite gels with 0.30% mechanically fibrillated (**B**), and 0.30% oxidized CNF (**C**) at 40 × magnification. SEM images of supercritically-dried gels of the same composition (**D**–**F**).

Investigation of the composite gels with microscopy revealed that the cellulose was homogeneously distributed in the alginate gel that appear as transparent in the light microscope (Figure 5). No phase separation was seen either by optical microscopy or scanning electron microscopy, which further indicates a good integration between the alginate and the cellulose fibrils. The oxidized CNF has a higher (negative) charge than the fibrils in the mechanically-fibrillated quality (Table A2 in Appendix A). Additionally, the cellulose fibrils differed in size as seen in Figure 5, Appendix A Table A2, and from [25]. Although the oxidized CNF sample contains a large fraction of nanosized fibrils (optically inactive in Figure 5), it contains a major fraction of residual fibres (Figure 5, Appendix A, Table A2). It is, thus, a potential for further improvements of the mechanical properties of the composite hydrogels by increasing the yield of the fibrillation. However, the oxidized fibrils are much thinner and more homogeneous than the mechanically-fibrillated fibrils. Previous studies on composite films based on Ca^2+^ crosslinked alginate and cellulose showed increasing mechanical properties by increasing the amount of the fibrils in the biocomposite and by decreasing the fibril size. However, the introduction of negative charges on the surface of the cellulose fibrils increased the mechanical crosslinking even further [30]. Hence, this suggests that the charges, more than the size of the fibrils, influence the mechanical properties of the composite material.

In SEM, all the samples, including the alginate sample, show tread-like structures caused by the drying step which is necessary before SEM imaging (Figure 5). This is commonly seen for alginate hydrogels also when using supercritical drying, intended to preserve the original structure in the samples [31]. No formation of honeycomb structure, which is typically formed when freeze-drying CNF-samples, is observed [32]. A structural difference is observed upon the addition of CNF where some larger fibre structures was observed, both for the mechanically-produced and oxidized CNF. Additionally, here, for both types of cellulose fibrils, the fibrils seem to be well-integrated with the alginate with no indication of phase separation.

### 3.5. Volume Stability and Calcium Binding

**Figure 6:**
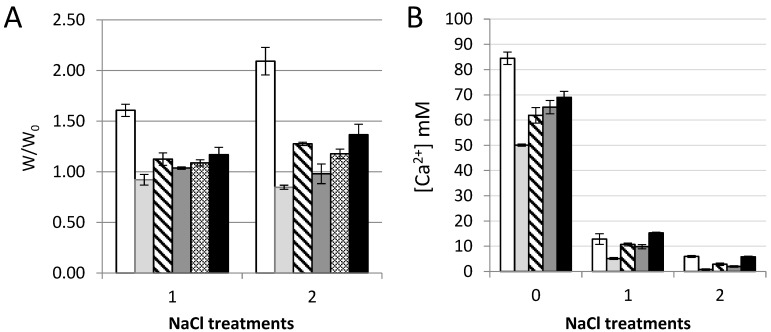
Calcium gels of *M. pyr*. alginate and oxidized CNF subjected to treatments with 0.9% (*w*/*v*) NaCl. Stability of gels (w/w_0_, (**A**)) and calcium concentration (**B**) before and after dialysis against 150 mM NaCl solution. 0.75% (*w*/*v*) *M. pyr*. alginate (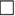), 0.75% (*w*/*v*) oxidized CNF (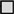), 0.375% (*w*/*v*) oxidized CNF + 0.375% (*w*/*v*) *M. pyr*. (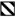), 0.75% (*w*/*v*) oxidized CNF + 0.2% (*w*/*v*) *M. pyr.* (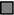), and 0.75% (*w*/*v*) oxidized CNF + 0.75% (*w*/*v*) *M. pyr*. (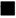). Mean ± standard deviation are shown for three gels.

The alginate and oxidized CNF gels were finally exposed to physiological saline solution (150 mM NaCl) to assess the volume stability and the binding of calcium in the gels (Figure 6). Surprisingly, the weak Ca-CNF gels (Figure 4) and all gels of alginate and oxidized CNF were stable during the exposure to NaCl solution. This is in contrast to the well-known swelling of Ca-alginate gels [33] recognized by the exchange of calcium ions with sodium ions and ingress of water into the gel. The stability in physiological solutions is important both for handling and in vitro studies of the biomaterial construct as well as for in vivo use. When the content of calcium was measured in the gels, an accumulation of calcium was found in the gels containing alginate for the Ca-saturated systems, with more calcium in the gels containing alginate solely than in the mixed system of same concentrations of alginate and oxidized CNF. Upon treatment with saline solution, increasing concentrations of calcium were found for increasing alginate concentrations. Similar calcium content was found in the gels containing alginate alone and alginate with oxidized CNF, pointing towards a more complex explanation of the increase in Young’s modulus (Figure 1, Figure 3, and Figure 4) than calcium binding solely.

### 3.6. Intermolecular Interaction Studied by FT-IR

**Figure 7:**
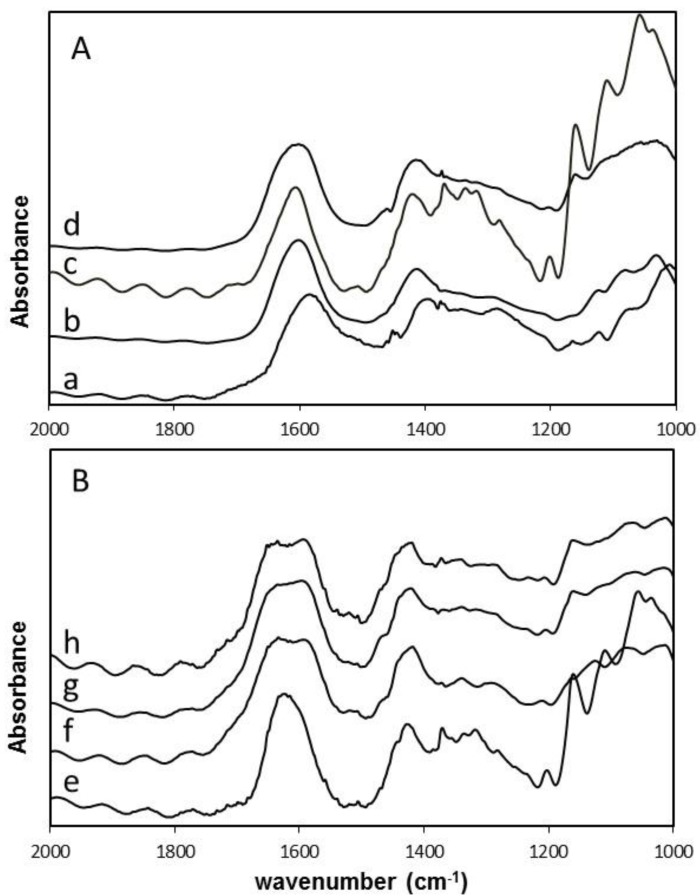
FT-IR spectra of freeze dried gel cylinders of 1.0% *M. pyr.* alginate and 0.75% CNF. (**A**) Calcium unsaturated samples and starting materials; (**B**) calcium saturated samples. *M. pyr.* powder, no calcium. (**a**); *M. pyr*, Ca-unsaturated (**b**); ox. CNF powder, no calcium (**c**); *M. pyr.* + ox CNF, Ca-unsaturated (**d**); Ox. CNF, Ca-saturated (**e**); *M. pyr.*, Ca-saturated (**f**); *M. Pyr.* + ox. CNF, Ca-saturated (**g**); and *M. Pyr.* + mechanically-fibrillated CNF, Ca-saturated (**h**).

FT-IR spectra of selected samples were obtained in an attempt to detect intermolecular interactions (Figure 7 and Appendix D). The spectra were normalized with respect to the characteristic ν(COO)_asym_ vibration band around 1600 cm^−1^. This was done for better comparison of the wavenumber region where calcium-polymer interaction is most likely to be observed. Both calcium-saturated and -unsaturated composite gels were studied to see if the calcium content influenced the carboxylic groups, as well as to compare our results to previous observations. Calcium-unsaturated gels contained only the calcium released from CaCO_3_(s) as a result of the hydrolysis of GDL during gel formation, whereas the calcium saturated gels were obtained after incubation in 50 mM CaCl_2_ + 200 mM NaCl for 24 h. As the spectra of the hydrogel composite itself are disturbed by the high water content, FTIR spectra of dried samples were obtained. Spectra of alginate powder and oxidized CNF powder were also compared with the dried CNF-alginate composite hydrogels.

It has previously been reported that the (C=O) stretching band from alginate is shifted from 1602 to 1612 cm^−1^ in oxidized CNF-alginate composite films with excess calcium removed. This has been attributed to the carboxylic groups on oxidized CNF linking alginate molecules to form a crosslinked network [34]. In the calcium-unsaturated samples we do not observe the same shift in signals. The calcium unsaturated samples gives ν(COO)_asym_ signals from 1602 to 1604 cm^−1^, for the pure alginate gel and for composite gel of alginate and oxidized CNF, respectively. The ν(COO)_asym_ signal from *M. pyrifera* alginate powder devoid of calcium is at 1583 cm^−1^ and oxidized CNF devoid of calcium is 1606 cm^−1^. The samples are, however, not directly comparable with [34], due to differences in sample preparation, charge density on oxidized CNF, fibril characteristics, alginate composition, and molecular weight. From our measurements, wedo not have strong evidence to support intermolecular interactions between oxidized CNF and *M. pyrifera* alginate mediated by calcium in the unsaturated calcium gels.

The calcium saturated composite samples were strikingly different, displaying a split in the carboxyl groups (C=O) stretching band around 1600 cm^−1^. This is not seen for the spectra of pure, calcium saturated oxidized CNF, but is observed for the pure alginate gel saturated with calcium. Our hypothesis is that the splitting of the bands for the alginate-containing samples is due to differences in Ca^2+^ binding of carboxylic acid groups from mannuronic acid and guluronic acid. The G-block junction zones will tend to associate laterally at high calcium concentrations [35], and this may shift the signal from guluronic acid towards higher wavenumbers while the carboxylic group in mannuronic acid does not bind calcium in a specific manner and, therefore, does not shift.

As a last comment one should be careful to extrapolate results obtained on dried composite films to composite hydrogels as there will be large differences in both polymer concentration and ionic strengths. It would, therefore, be desirable to be able to study molecular interactions with methods where water interference is not an issue.

## 4. Conclusions

This study shows that tightly integrated composite hydrogels with tailored mechanical properties can be made from alginate and nanofibrillated cellulose in calcium saturated gels. The alginate contributes with elastic properties and increased mechanical resistance at large deformations. The cellulose nanofibrils reduces the syneresis of the alginate gels and contributes to increased resistance against compression at small deformations as seen by an increase in Young’s modulus. The effect was increased using oxidized nanofibrils where composite gels also were volume stable upon saline treatments. No net increase in calcium concentration was found in the composite gels relative to the alginate gel alone. Additionally, no change in FT-IR spectra of the (C = O) stretching band was found for dried composite gels relative to dried alginate gel alone. Although no specific calcium binding event could be identified, we cannot rule out possible calcium mediated CNF-alginate or CNF-CNF interactions, as the oxidized cellulose fibrils by themselves form gels with calcium ions and as more than an additive effect on Young’s modulus was seen for the combined system. The increase in Young’s modulus was not seen when other relevant biopolymers were added to the alginate gel. Hence, mechanical properties of the composite hydrogels can be tailored by selecting alginates with varying block compositions and charge/concentration of cellulose fibrils. This is relevant for the use of this composite system in films and membranes in addition to the direct use of these composite hydrogels in, e.g., bioprinting and tissue engineering applications.

## Figures and Tables

**Table 1:** *M*_w_ and sequence parameters in alginates used in this study [20] ^1^.

Alginate	*M*_w_ (kDa)	PI	F_G_	F_M_	F_GG_	F_GM_	F_MM_	F_MGM_	F_GGG_	N_G>1_
*D. potatorum*	163	1.76	0.32	0.68	0.20	0.12	0.56	0.07	0.16	6
*M. pyrifera*	177	1.94	0.41	0.59	0.21	0.20	0.40	0.18	0.17	5
*L. hyperborea*	200	2.23	0.67	0.33	0.56	0.11	0.23	0.08	0.52	13

^1^ Molecular weight (*M*_w_) and polydispersity index (PI = *M*_w_/*M*_n_) were determined from SEC-MALLS. Sequence parameters were calculated from ^1^H-NMR spectra. F_G_ and F_M_ denotes the fraction of guluronic and mannuronic acid, respectively. Fractions of dimers and trimers of varying composition are denoted by two and three letters, respectively.

**Figure A1:**
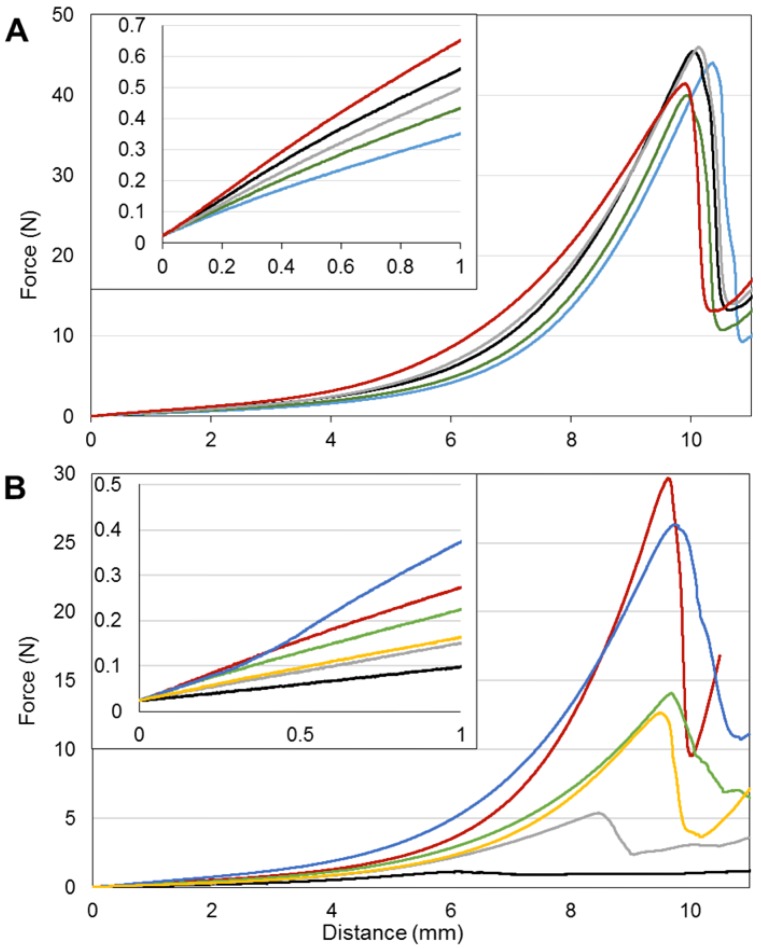
Stress-deformation curves of gel cylinders presented in Figure 1B and Figure 2B (**A**) and Figure 3 (**B**). The inserted picture shows the initial compression where the gels display elastic behaviour. Young’s modulus is calculated from 0.1 to 0.3 mm. (A) 1% (*w*/*v*) *M. pyr*. (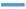), 1% (*w*/*v*) *M. pyr*. + 0.15% (*w*/*v*) ox. CNF (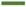), 1% (*w*/*v*) *M. pyr.* + 0.30% (*w*/*v*) ox. CNF (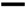), 1% (*w*/*v*) *M. pyr* + 0.50% (*w*/*v*) ox. CNF (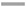) and 1% (*w*/*v*) *M. pyr* + 0.75% (*w*/*v*) ox. CNF (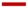); (B) 0.75% (*w*/*v*) *M. pyr* (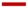), 0.75% (*w*/*v*) ox. CNF (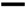), 0.375% (*w*/*v*) ox. CNF + 0.375% (*w*/*v*) *M. pyr* (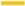), 0.75% (*w*/*v*) ox. CNF + 0.2% (*w*/*v*) *M. pyr* (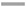), 0.75% (*w*/*v*) ox. CNF + 0.375% (*w*/*v*) *M. pyr* (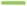) and 0.75% (*w*/*v*) ox. CNF + 0.75% (*w*/*v*) *M. pyr* (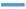).

## Appendix A

**Table A1:** Carbohydrate composition in the different CNF-samples.

Sample	Carbohydrate Composition (Weight %)				
	Glucan	Xylan	Mannan	Arabinan	Galactan
Softwood pulp	85.32	8.48	6.63	0.62	0.59
Mechanically fibrillated CNF	84.46	7.98	5.96	0.61	0.67
Oxidized CNF	66.25	6.63	2.67	0.03	0.00

The discrepancy in total monosaccharides between mechanically fibrillated CNF and oxidized CNF is due to conversion to uronic acids upon axidation in the oxidized sample, which were not quantified.

**Table A2:** Nanocellulose characteristics. All data, except residual fibres, are previously published in [25].

Sample	Charge Density (mmol/g)	Intrinsic Viscosity (mL/g)	Fibril Diameter (nm)	Fibril Length (μm)	Residual Fibres (%)
Mechanically fibrillated CNF	0.1	620	<100	>1	1.3
Oxidized CNF	0.9 ^1^	450	<20	>1	28.5

^1^ Aldehyde content was determined for the oxidized quality; 0.2 mmol/g CNF.

## Appendix B

**Table A3:** Statistical outcomes, Figure 1: E = Young’s modulus, standard deviation (SD), number of samples (*n*), and *p*-value (*p*) of samples compared to the respective pure alginate sample.

Sample	*n*	E (kPa), Mean	SD	*p* (%)	Syneresis (%), Mean	SD	*p* (%)
*D. pot.*	9	3.794	0.256	-	24.74	0.72	-
*0.15% CNF*	7	4.930	0.152	<0.01	22.95	0.79	0.08
*0.30% CNF*	6	5.134	0.399	<0.01	22.27	0.81	0.01
*0.50% CNF*	7	8.318	0.356	<0.01	15.60	1.01	<0.01
*0.75% CNF*	5	11.017	0.546	<0.01	13.53	1.11	<0.01
*0.15% ox. CNF*	7	5.036	0.272	<0.01	21.25	0.72	<0.01
*0.30% ox. CNF*	5	7.704	0.294	<0.01	17.78	0.50	<0.01
*0.50% ox. CNF*	6	12.786	0.549	<0.01	12.46	0.76	<0.01
*0.75% ox. CNF*	5	18.243	1.380	<0.01	7.17	1.45	<0.01
*M. pyr.*	7	*11.492*	*0.633*	*-*	*49.09*	*0.65*	*-*
*0.15% CNF*	8	14.895	0.743	<0.01	47.16	0.31	<0.01
*0.30% CNF*	7	17.437	1.259	<0.01	43.27	0.49	<0.01
*0.50% CNF*	5	18.273	0.779	<0.01	39.81	0.52	<0.01
*0.75% CNF*	5	24.297	2.950	<0.01	34.26	1.66	<0.01
*0.15% ox. CNF*	6	14.884	1.465	0.02	47.24	0.44	0.01
*0.30% ox. CNF*	5	19.210	1.504	<0.01	40.95	0.69	<0.01
*0.50% ox. CNF*	5	25.444	1.533	<0.01	31.83	2.20	<0.01
*0.75% ox. CNF*	5	36.060	3.666	<0.01	28.74	0.69	<0.01
*L. hyp.*	7	32.369	1.181	-	28.81	0.57	-
*0.30% CNF*	8	36.668	1.551	<0.01	27.26	0.87	<0.01
*0.75% CNF*	6	61.167	3.694	<0.01	20.20	0.42	<0.01
*0.30% ox. CNF*	8	42.991	1.970	<0.01	23.39	0.54	<0.01
*0.75% ox. CNF*	6	70.956	3.735	<0.01	15.82	1.18	<0.01

**Table A4:** Statistical outcomes, Figure 2: Rupture strength (mean), deformation (mean) standard deviation (SD), number of samples (*n*), and *p*-value (*p*) of samples compared to the respective pure alginate sample.

Sample	*n*	Rupture Strength (kg), Mean	SD	*p* (%)	Deformation (%), Mean	SD	*p* (%)
*D. pot.*	9	1.42	0.12	-	63.55	1.13	-
*0.15% CNF*	7	1.32	0.17	22.7	58.89	1.45	<0.01
*0.30% CNF*	6	1.23	0.14	2.45	57.70	1.39	<0.01
*0.50% CNF*	7	1.63	0.14	0.79	58.92	1.05	<0.01
*0.75% CNF*	5	1.66	0.11	0.35	58.67	0.96	<0.01
*0.15% ox. CNF*	7	1.15	0.03	0.02	55.70	0.60	<0.01
*0.30% ox. CNF*	5	1.35	0.02	19.1	56.56	0.35	<0.01
*0.50% ox. CNF*	6	1.59	0.05	0.79	55.44	0.61	<0.01
*0.75% ox. CNF*	5	1.39	0.09	66.3	51.63	1.35	<0.01
*M. pyr.*	*7*	4.09	0.56	-	71.76	1.09	-
*0.15% CNF*	8	5.50	0.42	0.03	71.40	1.02	50.3
*0.30% CNF*	7	5.31	0.20	0.03	69.27	1.08	0.11
*0.50% CNF*	5	5.15	0.30	0.35	69.25	0.62	0.06
*0.75% CNF*	5	5.43	0.31	0.07	66.99	0.68	<0.01
*0.15% ox. CNF*	6	4.16	0.07	75.4	67.41	0.38	<0.01
*0.30% ox. CNF*	5	4.07	0.04	95.2	63.99	0.34	<0.01
*0.50% ox. CNF*	5	4.54	0.22	12.1	62.96	0.59	<0.01
*0.75% ox. CNF*	5	4.25	0.17	55.0	59.71	0.67	<0.01
*L. hyp.*	7	5.11	0.81	-	64.38	1.89	-
*0.30% CNF*	8	4.25	0.15	<0.01	60.16	0.70	<0.01
*0.75% CNF*	6	5.38	0.45	<0.01	60.07	0.93	<0.01
*0.30% ox. CNF*	8	3.88	0.17	<0.01	57.18	0.81	<0.01
*0.75% ox. CNF*	6	4.38	0.41	<0.01	55.32	1.43	<0.01

**Table A5:** Statistical outcomes, Figure 4A,B: E = Young’s modulus, standard deviation (SD), number of samples (*n*), and *p*-value (*p*) of samples compared to the 1.0% alginate sample for control polymers. *P*-values for CNF (*) are comparisons to the respective dry weight concentrations of alginate (1.30% and 1.75%).

Sample	*n*	E (kPa)	SD	*p* (%)	Syneresis (%)	SD	*p* (%)
*1.0% M.pyr.*	7	11.493	633	-	49.09	0.65	-
*0.30% M.pyr.*	8	15.916	1.285	<0.01	43.22	0.29	<0.01
*0.30% dextran*	9	12.168	0.967	14.2	44.81	0.90	<0.01
*0.30% hyaluronan*	8	13.150	0.710	0.08	38.01	0.60	<0.01
*0.30% xanthan*	8	14.829	0.330	<0.01	38.59	0.80	<0.01
*0.75% M.pyr.*	8	22.773	1.092	<0.01	34.31	0.51	<0.01
*0.75% dextran*	9	12.314	0.560	2.05	41.77	0.61	<0.01
*0.75% hyaluronan*	9	12.674	2.555	26.3	23.42	1.65	<0.01
*0.75% xanthan*	9	19.483	1.237	<0.01	26.57	0.79	<0.01
*0.30% CNF**	7	17.437	1.259	4.37	43.27	0.49	81.9
*0.30% ox. CNF**	5	19.210	1.504	0.18	40.95	0.69	<0.01
*0.75% CNF**	5	24.297	2.950	20.7	34.26	1.66	94.0
*0.75% ox. CNF**	5	36.060	3.666	<0.01	28.74	0.69	<0.01

**Table A6:** Statistical outcomes, Figure 4C,D: Statistical outcomes, Figure 4: E = Young’s modulus, standard deviation (SD), number of samples (*n*), and *p*-value (*p*) of samples compared to the 1.0% alginate sample for other polymers CNF. *P*-values for CNF (*) are comparisons to the respective dry weight concentrations of alginate (1.30% and 1.75%).

Sample	*n*	Rupture Strength (kg), Mean	SD	*p* (%)	Deformation (%), Mean	SD	*p* (%)
*1.0% M.pyr.*	7	4.09	0.56	-	71.76	1.00	-
*0.30% M.pyr.*	8	5.99	0.29	<0.01	71.17	1.25	34.0
*0.30% dextran*	9	5.07	0.23	0.08	72.17	0.82	38.3
*0.30% hyaluronan*	8	3.69	0.60	21.0	74.86	3.87	6.76
*0.30% xanthan*	8	3.18	0.21	0.16	63.70	0.61	<0.01
*0.75% M.pyr.*	8	9.67	0.44	<0.01	69.74	1.24	0.64
*0.75% dextran*	9	4.81	0.41	1.37	70.79	1.31	13.6
*0.75% hyaluronan*	9	2.54	0.33	<0.01	64.02	1.78	<0.01
*0.75% xanthan*	9	2.81	0.08	<0.01	60.76	0.40	<0.01
*0.30% CNF**	7	5.31	0.20	0.04	69.27	1.08	1.06
0.30% ox. CNF*	5	4.07	0.04	<0.01	63.99	0.34	<0.01
0.75% CNF*	5	5.43	0.31	<0.01	66.99	0.68	0.015
0.75% ox. CNF*	5	4.25	0.17	<0.01	59.71	0.67	<0.01

## Appendix D

**Table A7:** FTIR peaks for ν(COO)_asym_ (cm^−1^) for samples shown in Figure 7.

	Sample	Calcium Saturated Gel	ν(COO)_asym_ (cm^−1^)
a	*M. pyr.* powder, no calcium	pristine	1583
b	1.0% (*w*/*v*) *M. pyr*	no	1602
c	ox. CNF, no calcium	pristine	1606
d	1% (*w*/*v*) *M. pyr* + 0.75% (*w*/*v*) ox. CNF	no	1604
e	0.75% (*w*/*v*) ox. CNF	yes	1624
f	1.0% (*w*/*v*) *M. pyr.*	yes	1589/1633 *
g	1.0% (*w*/*v*) *M. pyr*. + 0.75% (*w*/*v*) ox. CNF	yes	1593/1635 *
h	1.0% (*w*/*v*) *M. Pyr*. + 0.75% (*w*/*v*) mechanically fibrillated CNF	yes	1591/1639 *

* Two unresolved signals centered around 1620 cm^−1^.
